# 

**Published:** 1922-10

**Authors:** 


### HOSPITAL and HEALTH

REVIEW

*Established 1886* No. 1855. Vol. LXXI.

*New Series.* Vol. 1. No. 13 *October* 1922 Price Sixpence

CONTENTS.

| | PAGE |
|---|---|
| NOTES AND COMMENTS—<br>The Lochmaree Outbreak of Food Poisoning—Unhygienic Food Factories—Milk Orders—The Barnet Old Age Pension Case—The Nation in Statistics—Lead Poisoning from Beer—Doctors' Fees in Germany—Government Research—The Future of Sanatorium Industries—Factory Inspection and Welfare Work—The Fumigation of Ships—Labour and Housing—Antitoxin and Diphtheria—A Colour Scheme for Poisons—Hospitals and Sunday Games | 375 |
| THE SANITARY INSPECTOR—<br>Conference of Sanitary Inspectors' Association at Buxton: Presidential Address | 382 |
| The Smoke Nuisance | 385 |
| THE PUBLIC HEALTH: INTERVIEWS WITH LOCAL AUTHORITIES. IV.—THE CITY OF SHEFFIELD | 386 |
| THE WRONG TURNING IN PUBLIC HEALTH. By F. E. Wynne, M.A., B.Ch., D.P.H. | 388 |
| MEDICINES AND MEDDLESOMENESS.—I. By R. W. Harris | 391 |
| THE REPORT OF THE WAR OFFICE COMMITTEE ON "SHELL-SHOCK" | 393 |
| OUR PENAL AND PRISON SYSTEM.—II. | 394 |
| PHYSICAL EDUCATION FOR GIRLS | 397 |
| THE HOSPITAL MANAGEMENT AND TREATMENT OF INFECTIOUS DISEASES.—II. By A. E. Pearson, L.R.C.P., M.R.C.S. | 398 |
| THE HOSPITAL SATURDAY COMMITTEE. By F. Squires. | 400 |
| HOSPITAL FINANCE | 401 |
| GOVERNMENT PUBLICATIONS | 402 |
| HOSPITAL NEWS | 403 |
| CORRESPONDENCE—<br>The Work of the Panel Committees—Co-operation in a Contributory Scheme—Another Contributory Scheme | 404 |
| RECENT APPOINTMENTS | 405 |
| OFFICIAL REPORTS RECEIVED | 405 |
| THE ROYAL SANITARY INSTITUTE | 406 |
| LECTURES ON INFANT AND CHILD WELFARE | 406 |

